# A Case Report of Type B Aortic Dissection With Ruptured False Lumen

**DOI:** 10.7759/cureus.63959

**Published:** 2024-07-06

**Authors:** Pokhraj P Suthar, Mohamed Z Hussein, Ramya Gaddikeri, Palmi Shah

**Affiliations:** 1 Department of Diagnostic Radiology and Nuclear Medicine, Rush University Medical Center, Chicago, USA

**Keywords:** imaging, classification, chest, cta, aortic dissection

## Abstract

Aortic dissection is a critical condition characterized by an intimal tear in the aortic wall, leading to the formation of a false lumen. We present a case of a 54-year-old male with chronic type B aortic dissection and hypertension who presented with acute tearing left back pain. Initial evaluation revealed elevated blood pressure and subtle laboratory abnormalities. Imaging confirmed a Stanford type B aortic dissection with an intramural hematoma and contained rupture of the false lumen. Despite initial stabilization efforts, the patient deteriorated rapidly and succumbed. This case highlights the critical importance of early diagnosis.

## Introduction

Aortic dissection is a life-threatening condition characterized by the development of a tear in the intimal layer of the aorta, allowing blood to flow between its layers and creating a false lumen [1]. This process can lead to severe complications such as hemopericardium, cardiac tamponade, and sudden death [2,3]. The incidence of aortic dissection is relatively low compared to other cardiovascular emergencies but carries high morbidity and mortality rates, especially in cases involving the descending thoracic aorta (type B dissections) [4]. Chronic type B lesions, in particular, pose ongoing risks of aneurysmal dilation and rupture, necessitating vigilant monitoring and timely intervention. Diagnostic imaging, including computed tomography angiography (CTA), plays a crucial role in identifying and classifying dissections, guiding management decisions, and assessing for complications like false lumen rupture [5]. This report highlights a rare case of Stanford type B aortic dissection with a ruptured false lumen, emphasizing the need for prompt recognition and aggressive management to optimize patient outcomes.

## Case presentation

A 54-year-old male with a history of chronic type B aortic dissection and hypertension was presented in the emergency department for the acute onset of new tearing left back pain. He denied any shortness of breath, fever, chills, weight loss, recent trauma, or travel. His medical history included hypertension and smoking, and he was on antihypertensive medication. On examination, the patient was hypertensive with a blood pressure of 156/98 mmHg. Other vital signs included a temperature of 100.1°F, a heart rate of 83 bpm, a respiratory rate of 18 breaths per minute, and an oxygen saturation of 99% on room air. Cardiovascular examination revealed a normal rate and regular rhythm. Carotid and radial pulses were 2+ bilaterally, and heart sounds were normal with no murmurs. The remainder of the physical examination was within normal limits.

Laboratory findings included an elevated lactate level, an unremarkable complete metabolic panel (CMP) with normal magnesium and phosphate levels, borderline leukocytosis, a normal hemoglobin level, and an adequate platelet count (Table 1). Electrocardiogram (EKG) findings indicated normal sinus rhythm with left axis deviation.

**Table 1:** Laboratory work-up eGFR: estimated glomerular filtration rate; RSV: respiratory syncytial virus; SARS-CoV-2: severe acute respiratory syndrome coronavirus 2

Parameters	Laboratory values	Reference ranges
White blood count	10.62 K/uL	4.00-10.00 K/uL
Red blood count	6.14 M/uL	4.40-6.40 M/uL
Hemoglobin	13.5 g/dL	12.0-18.0 g/dL
Platelet count	399 K/uL	150-450 K/uL
Sodium	137 mmol/L	137-147 mmol/L
Potassium	4.5 mmol/L	3.4-5.3 mmol/L
Chloride	101 mmol/ L	99-108 mmol/ L
CO_2_ total	24 mmol/L	22-29 mmol/L
Blood urea nitrogen (BUN)	11 mg/dL	8-21 mg/dL
Creatinine	0.96 mg/dL	0.75-1.20 mg/dL
eGFR	89 mL/min/1.73 sq m.	>=90 mL/min/1.73sq m.
Glucose	92 mg/dL	60-99 mg/dL
Calcium	10.1 mg/dL	8.7-10.7 mg/dL
High-sensitivity troponin I	7.8 ng/mL	0-35 ng/mL
Flu/RSV/SARS-CoV-2 panel	Not detected	Not detected
Lactic acid arterial	11.9 mmol/L	0.4-1.3 mmol/L
Magnesium	1.9 mg/dL	1.6-2.7 mg/dL
Phosphorus	3.0 mg/dL	2.5-4.6 mg/dL

**Figure 1:**
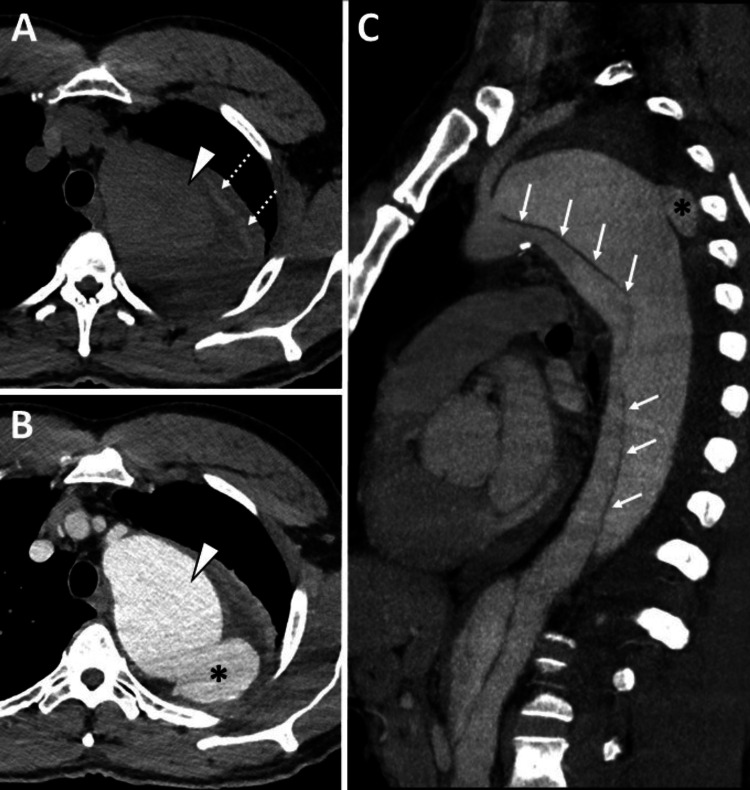
CT angiography of the chest was performed after the administration of 80 mL of intravenous contrast iopamidol 76% (Isovue 370, Bracco) in the right upper extremity. (A) Unenhanced axial CT, (B) contrast-enhanced axial CT, and (C) oblique aorta MIP (maximum intensity projection) demonstrate a type B aortic dissection with the dissection flap extending distally from the origin of the left subclavian artery (solid white arrows in C). Additionally, there is an intramural hematoma along the proximal descending thoracic aorta (dashed white arrow in A) and a localized saccular outpouching indicating contained rupture (black asterisk in B and C) from the posterior false lumen of the proximal descending thoracic aortic dissection with aneurysmal dilation (white arrowhead in B).

**Figure 2:**
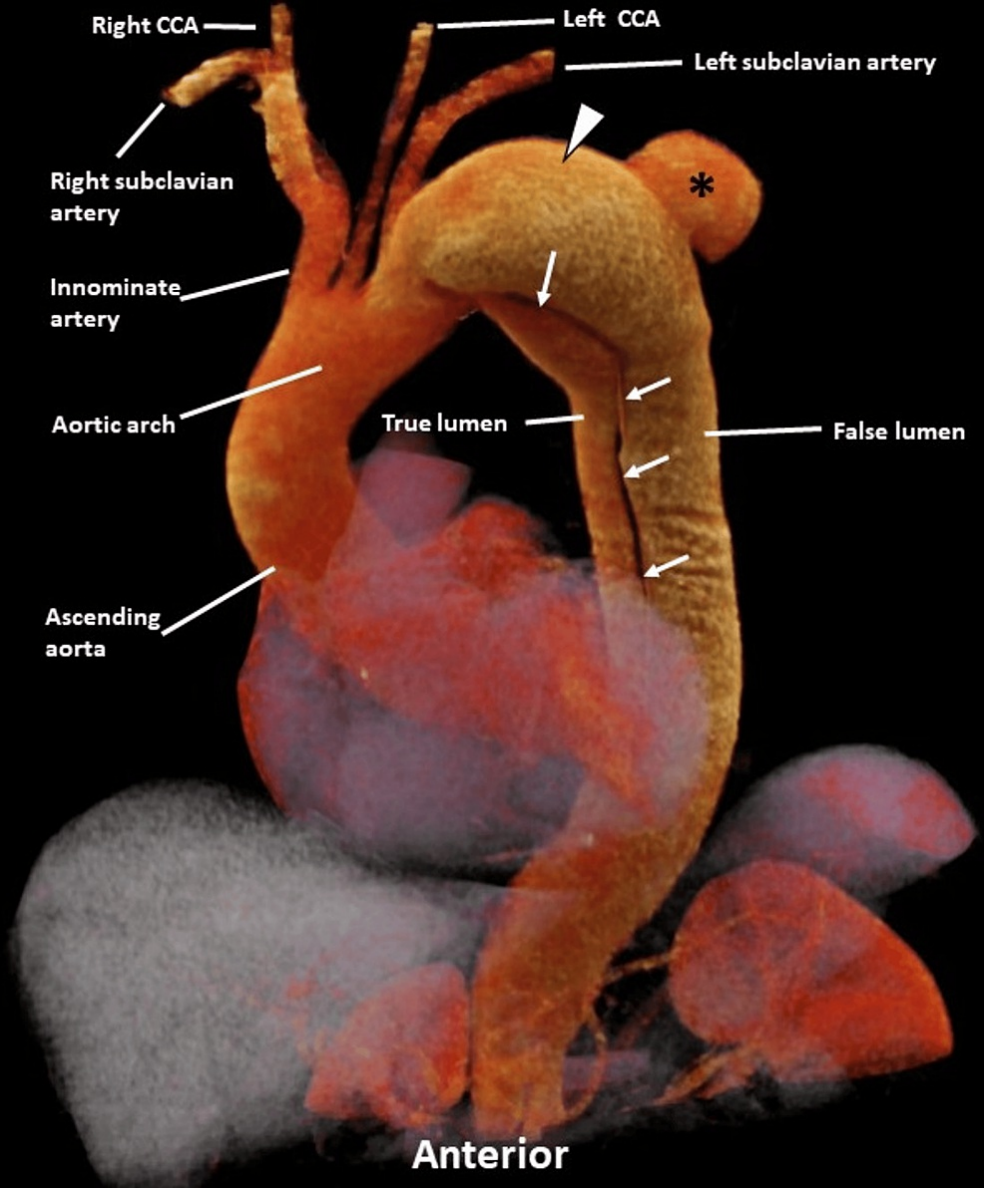
A three-dimensional cinematic volume-rendered image (SIEMENS Healthineers, Erlangen, Germany) of CT angiography of the chest following the injection of 80 mL of intravenous contrast iopamidol 76% (Isovue 370, Bracco) into the right upper extremity reveals a type B aortic dissection. The dissection flap extends distally from the origin of the left subclavian artery (solid white arrows). There is also a localized saccular outpouching indicating contained rupture (black asterisk) from the posterior false lumen of the proximal descending thoracic aortic dissection, accompanied by aneurysmal dilation (white arrowhead). CCA: common carotid artery

To manage his hypertension, the patient was administered esmolol and labetalol. A CTA of the chest was performed, revealing a Stanford type B aortic dissection with a dissection flap extending distally from the origin of the left subclavian artery. Additionally, an intramural hematoma was observed along the proximal descending thoracic aorta. The imaging also showed a localized saccular outpouching indicative of a contained rupture from the posterior false lumen of the proximal descending thoracic aortic dissection, along with aneurysmal dilation and a surrounding periaortic hematoma (Figures 1, 2).

After stabilization, the patient was transported to the cardiovascular intensive care unit (CICU). Upon arrival, his vital signs were as follows: BP 176/126 mmHg, heart rate 90 bpm, respiratory rate 19 breaths per minute, oxygen saturation 97% on room air, and he was afebrile. The physical exam was unremarkable. The patient was alert and oriented to person, place, time, and situation, but continued to endorse mild yet unrelenting chest pressure and back pain. Approximately 20 minutes after arriving in the CICU, the patient became unresponsive while speaking with the bedside staff. The CICU team responded immediately and found the patient slumped over with agonal breathing and an associated drop in blood pressure. Despite several rounds of cardiopulmonary resuscitation (CPR), the patient was unable to survive and ultimately succumbed.

## Discussion

Aortic dissection is an urgent medical condition characterized by a tear in the inner layer of the aorta, which allows blood to flow between the layers of the aortic wall, and form a false lumen. This process can result in a significant decrease in systemic blood pressure and may lead to complications such as hemopericardium and cardiac tamponade, potentially culminating in sudden death [1]. The incidence of aortic dissection is 0.5 to 3 cases per 100,000 people per year. Approximately 75% of aortic dissections occur in individuals aged 40 to 70 years, and the majority occurring between 50 and 65 years [6].

The normal aorta is composed of collagen, elastin, and smooth muscle cells, which form its layers: the intima, media, and adventitia. With aging, degenerative changes cause the breakdown of collagen, elastin, and smooth muscle, along with an increase in basophilic ground substance. This process is known as cystic medial necrosis, a characteristic histologic change associated with aortic dissection and Marfan syndrome. Risk factors for aortic dissection include aging, atherosclerosis, blunt trauma, cocaine use, coarctation of the aorta, aortopathy (e.g., Marfan syndrome, Ehlers-Danlos syndrome), metabolic disorders (e.g., homocystinuria), bicuspid aortic valve, connective tissue disorders, infectious or inflammatory conditions (e.g., vasculitis, tertiary syphilis), previous heart surgery, and pregnancy [7].

The duration of aortic dissection is typically divided into three phases: acute (within the first 14 days of symptom onset), subacute (between 14 days and three months), and chronic (more than three months after the initial symptoms appear) [8]. Patients with aortic dissection are often hypertensive (though they can be normotensive or hypotensive) and present with chest pain and a tearing sensation, with possible blood pressure differences between arms. End-organ ischemia (up to 27% of cases) may affect abdominal organs, limbs, brain (stroke), and spinal cord (paraplegia) [2]. If the aortic root is involved, it can mimic ST-elevation myocardial infarction on an EKG, but antiplatelets/anticoagulation can be disastrous. Rupture can cause collapse, often leading to death and cardiac tamponade. The aortic dissection detection risk score (ADD-RS) and a negative D-dimer test can help reduce unnecessary exams but need further validation [3,9].

Imaging is crucial for assessing the morphology and extent of aortic dissection, enabling classification, which determines management, with the Stanford and DeBakey classification systems commonly used to categorize dissections based on ascending aorta involvement. The Stanford classification categorizes dissections based on their proximal involvement, where type A affects any part of the aorta proximal to the origin of the right brachiocephalic trunk, while type B occurs distal to this point [10]. Chronic type B lesions continue to have considerable morbidity and mortality [4].

Imaging is crucial for evaluating and classifying thoracic aortic dissection. Chest radiography may reveal signs such as a widened mediastinum, double aortic contour, irregular aortic shape, and displacement of atherosclerotic calcification [11]. CT, especially with arterial contrast (CTA), is the preferred diagnostic tool due to its high sensitivity and specificity in identifying and categorizing dissections, detecting complications, and visualizing subtle findings like high-density mural hematoma and displaced calcification [5]. Magnetic resonance imaging (MRI) and transesophageal echocardiography (TOE) are also valuable but less frequently utilized. Conventional angiography remains essential for endoluminal repair but has largely been supplanted by CTA for initial assessment, given its non-invasive nature and superior ability to visualize dissection details and associated complications.

Complications of aortic dissection include branch vessel dissection, abdominal organ ischemia (especially the left kidney), limb ischemia, ischemic stroke, paraplegia (involving the artery of Adamkiewicz), distal thromboembolism, aneurysmal dilatation requiring intervention, aortic rupture, and hemothorax. Stanford type A dissections can also lead to coronary artery occlusion (commonly the right coronary artery), aortic insufficiency, rupture into the pericardial sac causing hemopericardium and cardiac tamponade, pulmonary artery intramural hematoma, and acute obstructive right heart failure due to hematoma compression of the pulmonary arteries [6,12]. We only found one reported case in the literature describing rupture of the false lumen in aortic dissection [13].

Therapeutic options for dissecting abdominal aortic aneurysms include prosthetic replacement of the affected aorta, with treatment strategies similar to those used for atherosclerotic aneurysms. When feasible, endovascular techniques offer a less invasive alternative to surgery and are recommended in such cases [14].

## Conclusions

Acute aortic dissection is a subtype of acute aortic syndromes (AASs), which is characterized by abrupt acute severe tearing chest or back pain. Chronic type B lesions continue to have considerable morbidity and mortality. Diagnostic imaging is important for follow-up and monitoring. Persistent false lumen expansion and perfusion in type B lesions is a concern for aneurysms and ruptures, which require surgical repair or thoracic endovascular aortic repair (TEVAR). This case raises awareness of this rare entity and demonstrates a good prognostic outcome if diagnosed early.
